# Study of association between corneal shape parameters and axial length elongation during orthokeratology using image-pro plus software

**DOI:** 10.1186/s12886-024-03398-6

**Published:** 2024-04-12

**Authors:** W. Wang, J. Deng, C. Yin, F. Wang, C. Zhang, C. Yu, S. Gong, X. Zhan, S. Chen, D. Shen

**Affiliations:** 1 Hangzhou Xihu Zhijiang Eye Hospital, Hangzhou, China; 2 School of Ophthalmology and Eye Hospital, Wenzhou Medical University, Wenzhou, Zhejiang China

**Keywords:** Axial length elongation, Myopia control, Orthokeratology, Image-pro plus

## Abstract

**Background:**

The aim was to validate the correlation between corneal shape parameters and axial length growth (ALG) during orthokeratology using Image-Pro Plus (IPP) 6.0 software.

**Methods:**

This retrospective study used medical records of myopic children aged 8–13 years (*n* = 104) undergoing orthokeratology. Their corneal topography and axial length were measured at baseline and subsequent follow-ups after lens wear. Corneal shape parameters, including the treatment zone (TZ) area, TZ diameter, TZ fractal dimension, TZ radius ratio, eccentric distance, pupil area, and pupillary peripheral steepened zone(PSZ) area, were measured using IPP software. The impact of corneal shape parameters at 3 months post-orthokeratology visit on 1.5-year ALG was evaluated using multivariate linear regression analysis.

**Results:**

ALG exhibited significant associations with age, TZ area, TZ diameter, TZ fractal dimension, and eccentric distance on univariate linear regression analysis. Multivariate regression analysis identified age, TZ area, and eccentric distance as significantly correlated with ALG (all *P* < 0.01), with eccentric distance showing the strongest correlation (β = −0.370). The regressive equation was y = 1.870 − 0.235a + 0.276b − 0.370c, where *y* represents ALG, a represents age, b represents TZ area, and c represents eccentric distance; *R*^2^ = 0.27). No significant relationships were observed between the TZ radius ratio, pupillary PSZ area, and ALG.

**Conclusions:**

IPP software proves effective in capturing precise corneal shape parameters after orthokeratology. Eccentric distance, rather than age or the TZ area, significantly influences ALG retardation.

## Introduction

The global rise in myopia prevalence has drawn increased attention [1–3] to myopia management strategies, with orthokeratology emerging as a prominent approach [4–10]. Orthokeratology lenses temporarily re-shape the cornea to correct refractive errors and slow axial length progression delay by approximately 31–63% [1, 9, 11].

However, there exists considerable individual variability in efficacy of orthokeratology for myopia control. As orthokeratology becomes more prevalent, there is a growing urgency among optometrists to predict its effects conveniently and precisely. Previous studies suggest a correlation between axial length growth (ALG) and corneal morphology following orthokeratology [7, 8, 12–16]. However, accurately measuring parameters like the treatment zone (TZ) and pupillary peripheral steepened zone (PSZ) remains challenging [17]. Most studies rely on simulating the plastic zone as a regular circle or ellipse [7, 18, 19], leading to potential biases.

Image-Pro Plus (IPP) software offers advanced image analysis capabilities, enabling precise delineation of specific regions like the TZ and comprehensive data collection [17, 20, 21]. Building upon the work by Mei et al. [17] regarding the reliability of IPP in evaluating corneal reshaping with orthokeratology, this study used IPP software to investigate how corneal reshaping influences myopia control efficacy.

## Subjects and methods

### Ethical approval

This study received approval from Xihu Zhijiang Ophthalmology Hospital’s Ethics Committee (number: 2020-001-k-01-01) and adhered to the principles outlined in the Declaration of Helsinki.

### Subjects

Patients who underwent orthokeratology treatment with ortho-k lenses for 18 months at Xihu Zhijiang Ophthalmology Hospital between January 2017 and November 2019.

A total of 104 subjects (41 males and 63 females), aged 8–13 years, were retrospectively included. Inclusion criteria encompassed a spherical refractive error < − 6.00 DS and refractive astigmatism ≤ − 2.00 DC; best-corrected visual acuity of ≥ 20/20; no history of treatment for binocular vision disorder or vision training; contraindications to wearing contact lens; and no ocular pathology, strabismus, amblyopia, or other vision impairments.

### Lens fitting

All patients enrolled were prescribed four-zone reverse-geometry lenses with a total lens diameter ranging from 10.2 to 11 mm, a back optical zone diameter of 6.0–6.2 mm (conventional), a reverse curve width of 0.5 mm, an alignment curve between 1.0 and 1.4 mm, and a peripheral curve width of 0.5 mm. The VST products used were Alpha (nominal Dk, 104 × 10^11^ (cm^2^/s) (mLO_2_/mL×mmHg); Nagoya, Japan), Dreamlite (nominal Dk, 100 × 10^11^ (cm^2^/s) (mLO_2_/mL×mmHg); Gelderland, The Netherlands), DreamVision (nominal Dk, 100 × 10^11^ (cm^2^/s) (mLO_2_/mL×mmHg); AnHui, China), and Euclid (nominal Dk, 127 × 10^11^ (cm^2^/s) (mLO_2_/mL×mmHg); Virginia, USA)The final order was determined by adding 0.75 diopters D to the target power through over-refraction.

### Data collection

The clinical records of 104 subjects were retrospectively collected for this study, including data on patients’ age at initial orthokeratology lens fitting, sex, and baseline spherical equivalent (SE) refractive error, which was calculated as the sum of spherical power and half the cylindrical power.

### Measurements

After adequate mydriasis using topical 0.5% tropicamide,Retinoscopy was employed using a computer optometry instrument (Japan, NIDEK, ARK-510 A) to determine SE.

At baseline and subsequent follow-up visits, corneal topography (E300 Topographer, Medmont) was conducted, with a minimum of three topographic maps obtained at each measurement session. The highest-quality map was subsequently selected for analysis. From these maps, corneal topographic characteristics, and pupil size, were derived.

The study used a noncontact biometer (IOL Master; Carl Zeiss Jena GmbH, Jena, Germany) to measure baseline and 1.5-year axial length data. Three measurements were obtained at each visit, and the mean average value of the three separate measurements of axial length was recorded.

### Image analysis

IPP software was used to analyze the TZ and PSZ parameters.

### TZ parameters

The best topographical maps at baseline and the 3-month follow-up were chosen to generate composite tangential subtractive maps. Using custom settings, the step size of each tangential subtractive map was set to the minimum (0.1 D). These maps were imported into IPP software for analysis. TZ was determined following the methodology outlined by Mei Ying et al. [17], with a tolerance of ± 0.05 D for each zero point at the edge of the TZ. The boundary was depicted, and a reference for the analysis of TZ parameters using IPP is provided.

1. *TZ area*: Area of TZ.
2. *TZ diameter*: Diameter of the “zero diopter change” zone, including the maximum, minimum, and mean diameters.
3. *TZ fractal dimension*: The fractal dimension is a non-integer value describing the complexity of a fractal object that exhibits scale invariance. While Euclidean geometry assigns dimensions of 1 to lines, 2 to planes, and 3 to cubes, fractals can have dimensions between those values, such as 1.50 or 2.33, indicating fractional spatial dimensions. The outline of the TZ can be considered more dimensional than a line (1-dimensional) but less so than a square (2-dimensional), thus having a dimension between 1 and 2. A higher number reflects greater complexity in morphology [22, 23].
4. *TZ radius ratio*: The ratio between the maximum and minimum radius of the “zero diopter change” zone.
5. *Eccentric distance*: The distance between the center of TZ and the pupil center. The center of TZ was automatically displayed when the “Outline Style” of the “Count/Size Options” was set to “Dot,” and the pupillary center was determined via corneal topography.

### PSZ parameter

**Fig. 1:**
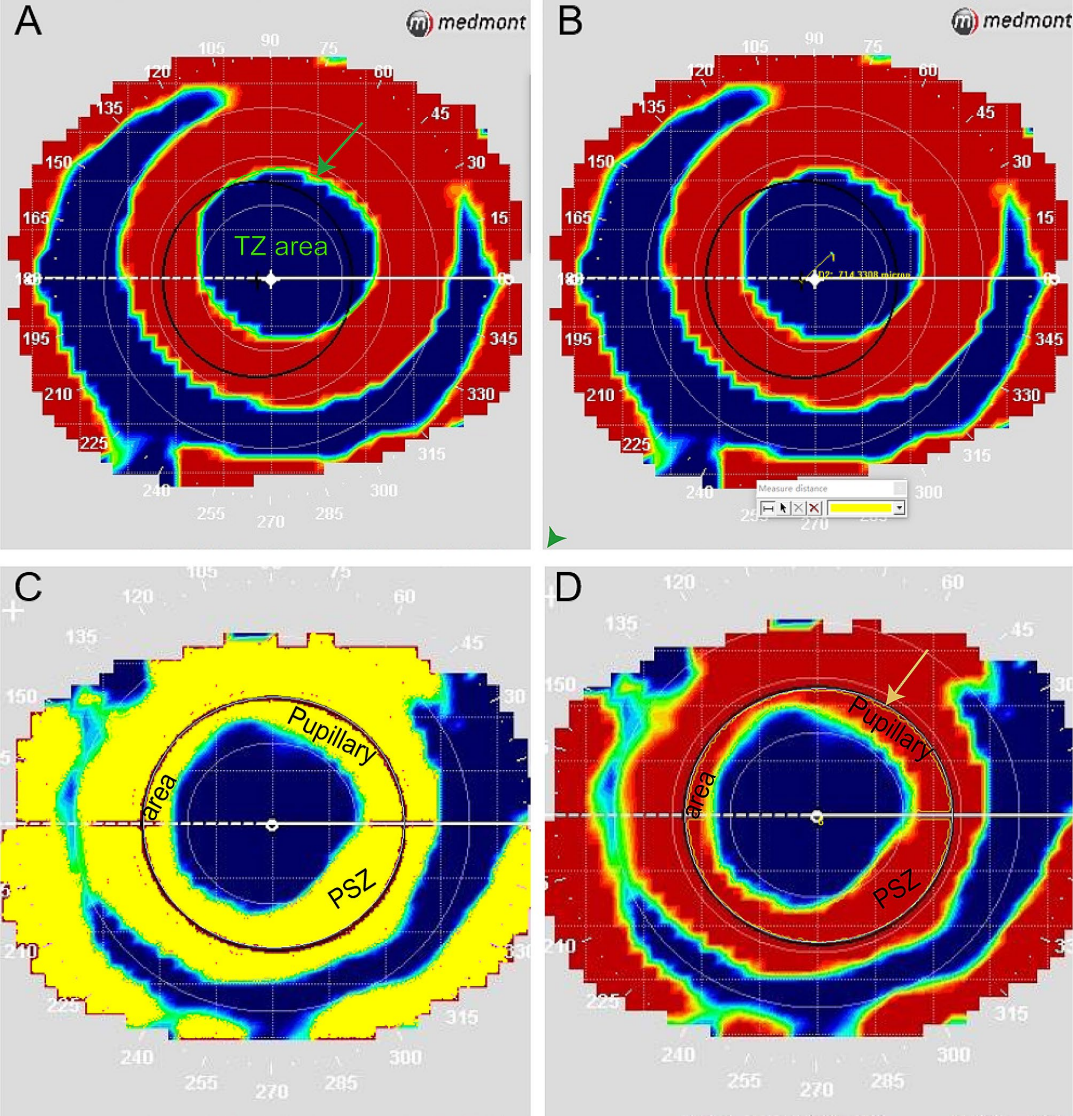
Image-Pro Plus software was used to evaluate the corneal shape parameters. **(A)** The treatment zone (TZ) area is manually delineated in green. **(B)** Manual measuring of the eccentric distance. **(C)** The peripheral steepened zone (PSZ) area is represented in yellow. **(D)** The pupillary PSZ area is delineated in yellow

1. *Pupil area*: The area of pupil.
2. *Pupillary PSZ area*: The PSZ of the pupil area.
  1. Preparation: Select the measurement scale and click “Measure→Count/Size→Options→Outline style→Outline” to show the outline of the actual measurement area.
  2. The pupil areas were depicted manually using “Ellipse AOI.”
  3. Click “Measure→Count/Size→Select Colors,” choose the straw-shaped button, and then put “the straw” on the red area (PSZ). Then, Click “Measure→Count/Size→Count,” which will display the contour line of the pupillary PSZ area (Fig. 1).
  4. Click “Measure→Select measurement→Area.”
3. *Pupillary PSZ area/pupil area*: The proportion of PSZ in the pupil area.

### Statistical analysis

The analyses were conducted using SPSS. A paired t-test compared measurements at 1.5 years after orthokeratology to baseline measurements. Associations between ALG and changes in age, SE, eccentric distance, TZ area, TZ diameter, TZ fractal dimension, TZ radius ratio, and pupillary PSZ area were assessed using Pearson correlation coefficients. Significant predictors of ALG were identified through multiple linear regression analysis, employing forward selection. Statistical significance was set at α = 0.05.

## Results

### Measurements

The mean age of participants was 10.69 ± 1.38 years. Baseline measurements revealed a mean SE of − 2.71 ± 1.13 D (range: −5.875 to − 0.50 D) and mean axial length of 24.71 ± 0.69 mm. Table 1 presents the corneal reshaping parameters.

**Table 1:** Corneal reshaping parameters

	Mean	SD
Eccentric distance (mm)	0.5	0.2
TZ area (mm^2^)	9.45	1.39
TZ diameter-max (mm)	3.68	0.27
TZ diameter-mean (mm)	3.42	0.26
TZ diameter-min (mm)	3.17	0.3
TZ Fractal dimension	1.04	0.01
TZ radius ratio	1.27	0.14
Pupil area	17.86	4.95
Pupillary PSZ area (mm^2^)	7.24	3.81
Pupillary PSZ area/Pupil area	0.37	0.12

### Axial growth VS. baseline characteristics

**Fig. 2:**
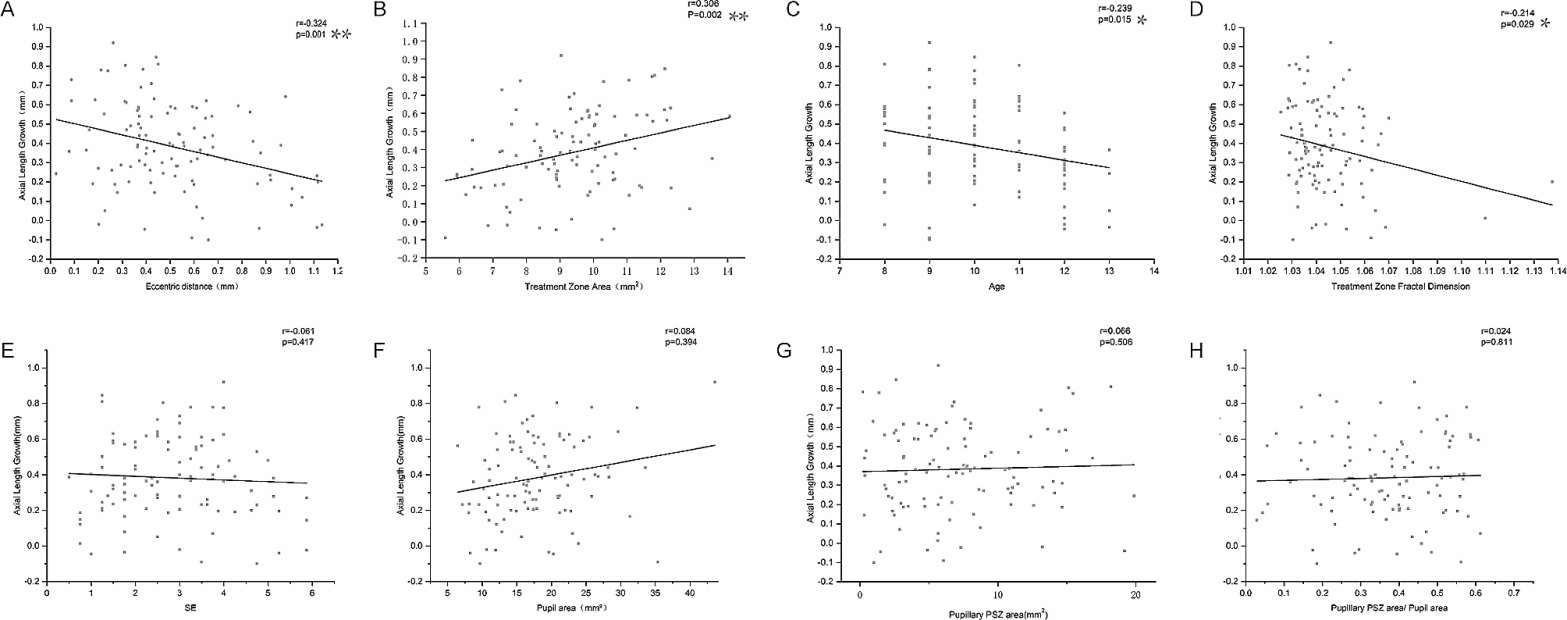
Scatterplots showing the correlation between axial length growth and **(A)** eccentric distance, **(B)** treatment zone (TZ) area, **(C)** age, **(D)** treatment zone (TZ) fractal dimension, **(E)** spherical equivalent (SE), **(F)** pupil area, **(G)** pupillary peripheral steepened zone (PSZ) area, and **(H)** pupillary PSZ area. (Pearson correlation (r) test; *P < 0.05; **P < 0.01)

At 18 months post-lens wear, the mean ALG was 0.38 ± 0.18 mm. Univariate regression analysis indicated a negative association between ALG and age (*P* = 0.015; Fig. 2C), while no correlation was observed with SE (*P* = 0.417; Fig. 2E). Multiple regression analysis demonstrated a significant association between age and ALG (Table 2).

**Table 2:** Linear regression analysis of the associations of 18-month ALG changes with various characteristics

	**Univariate model**		**Multivariate model**	
	***P Value***	**Standard Regression**	***P Value***	**Standard Regression**
		**Coefficient**		**Coefficient**
Age (years)	0.015	−0.239*	0.007	−0.235*
Baseline SE(D)	0.417	−0.061		
Eccentric distance	0.001	−0.324*	< 0.001	−0.370*
TZ area	0.002	0.306*	0.006	0.276*
TZ diameter-max	0.005	0.271*		
TZ diameter-mean	0.001	0.315*		
TZ diameter-min	0.002	0.306*		
TZ fractal dimension	0.029	−0.214*	0.385	−0.086
TZ radius ratio	0.145	−0.144		
Pupil area	0.394	0.084		
Pupillary PSZ area	0.506	0.066		
Pupillary PSZ area/Pupil area	0.811	0.024		

### Axial growth VS. cornea reshaping parameters

The relationship between ALG and corneal reshaping parameters is summarized in Table 2. ALG exhibited significant simple correlations with eccentric distance (Pearson correlation coefficient; *r* = − 0.324, *P* = 0.001; Fig. 2A), TZ area (*r* = 0.306, *P* = 0.002; Fig. 2B), TZ diameter—max (*r* = 0.271, *P* = 0.005), diameter—mean (*r* = 0.315, *P* = 0.001), TZ diameter—min (*r* = 0.306, *P* = 0.02), and TZ fractal dimension (*r* = − 0.214, *P* = 0.029; Fig. 2D). However, no significant correlations were found with changes in the TZ radius—ratio (*r* = − 0.144, *P* = 0.145), pupil area (*r* = 0.084, *P* = 0.394; Fig. 2F), pupillary PSZ area (*r* = 0.066, *P* = 0.506; Fig. 2G), and pupillary PSZ area/pupil area (*r* = 0.024, *P* = 0.811; Fig. 2H).

Given the calculation relationship between the TZ area and TZ diameter (max/mean/min), only the TZ area was included in the multivariate analysis. Multiple linear regression analysis results revealed significant correlations between ALG and changes in age, TZ area, and eccentric distance. Notably, the change in eccentric distance emerged as the most influential variable (β = −0.370, *P* < 0.0001). The regressive equation was y = 1.870 − 0.235a + 0.276b − 0.370c, where *y* is ALG, a is age, b is the TZ area, and c is the eccentric distance; *R*^2^ = 0.27. TZ fractal dimension did not contribute significantly to the model (*r* = − 0.086, *P* = 0.385; Table 2).

## Discussion

This study, employing IPP software, reported that children with a larger eccentric distance and smaller TZ area following orthokeratology exhibited a slower ALG rate at 18 months. Multiple regression analysis revealed significant associations between ALG and the initial age, eccentric distance, and TZD.

### Initial age and SE

Previous literature has consistently highlighted initial age as significant determinant of ALG [6, 8, 13, 18, 24–26]. Our findings align with this consensus, indicating a negative correlation between ALG and baseline age (*r* = − 0.247, *P* = 0.048). This suggests that myopia progression tends to decelerate with age [26].

Regarding baseline SE, our study did not detect a clear correlation with ALG, consistent with observations in some previous works [25–27]. However, some other studies have reported this association to be statistically significant [8, 18, 28]. Notably, studies have shown a significant negative correlation between ALG and SE in patients with a broader baseline SE range, typically between − 6.0 and − 1.0 D. Conversely, studies involving subjects with a narrower SE range, primarily between − 4.0 and − 1.0 D, tend to report no association between baseline SE and ALG [18]. In our study, the average SE was − 2.71 ± 1.13 D, with a higher proportion of cases with low SE (91.35% with < − 4.0 D), possibly contributing to the relatively rapid progression of myopia observed. Additionally, the inclusion of patients with potentially significant deviations in orthokeratology lens fitting may have influenced the myopia control effects associated with SE, thereby affecting the statistical outcomes.

### TZ parameters

Previous research by Kang et al. [29] indicated that orthokeratology induces changes in the central corneal power, leading to peri-retinal myopic defocusing and increased central foveal choroid thickness; this phenomenon is associated with a deceleration in ALG and myopia progression. Furthermore, Hu et al. [15] have demonstrated a strong negative correlation between changes in corneal refractive power within a 4-mm central diameter and myopia control efficacy induced by orthokeratology. Similar findings have been corroborated by other clinical investigations [12, 25, 30], supporting the hypothesis that a smaller TZ exposes a larger area to peripheral refraction, thereby potentially slowing myopia progression [7, 12, 25, 30, 31].

Previous studies have also suggested that orthokeratology-induced higher-order aberrations, including positive spherical [32] and coma-like [33] aberrations, may contribute to its myopia control effect [25]. Additionally, decentered orthokeratology has been associated with significant induction of additional corneal coma-like aberrations [34–36]. Notably, the decentration distance has been proposed as a predictive factor for myopia control efficacy following orthokeratology [7, 8, 28].

In our study, we found a positive association between the TZ area and ALG, while eccentric distance showed a negative correlation with ALG. These results align with those of other works, with eccentric distance exhibiting a stronger correlation with ALG. This observation is consistent with the findings of Hiraoka et al. [33], who emphasized the greater relevance of differences in coma-like aberrations to ALG than differences in positive spherical aberrations. This information may assist optometrists in more effectively adjusting treatment parameters and considering a more permissive approach to orthokeratology decentration without compromising visual acuity and patient comfort. Interestingly, we found no significant correlation between the TZ radius ratio and ALG, suggesting that pursuit of a perfectly circular TZ may be unnecessary.

The fractal dimension, an important parameter in fractal geometric analysis, has been widely described in various medical subfields [22, 23] but has not been extensively utilized in corneal measurement. Our study found that a larger fractal dimension was associated with slower ALG in univariate regression analysis. However, after adjusting for baseline age, eccentric distance, and TZ area, this association became statistically insignificant. These results indicate insufficient evidence to support the consideration of TZ fractal dimension as a relevant factor affecting ALG.

### PSZ parameter

In 2012, Chen et al. [37] reported that large scotopic pupil diameters (measured using OPD-ScanII) enhance the ALG slowing efficacy of orthokeratology in myopia (r^2^ = 0.405, *P* < 0.001). They speculated that a larger pupil diameter enhances the myopic shift in the peripheral retina, exerting a greater suppressive effect on ALG. These findings were confirmed by Miguel et al. [32] who reported that increasing pupil size, from 3 to 6 mm, led to higher exposure to peripheral defocusing. Similarly, recent research found significant correlations between ALG and the pupil area measured using corneal topography [8]. Thus, effective defocusing within the pupil area may aid in predicting the myopia control effect of orthokeratology lens fitting [26]. Despite these findings, some studies have reported no correlation between pupil diameter and the effectiveness of myopia control [26, 38].

Surprisingly, we observed no correlation between ALG and the pupil area, pupillary PSZ area, pupillary PSZ area/pupil area, suggesting that a larger pupillary PSZ area in the cornea may not reliably indicate better myopic control. We speculate several reasons for this discrepancy. First, there may be errors in pupil size measurements obtained via corneal topography compared with those obtained using an infrared pupillary detector, leading to differences in pupillary PSZ area estimates. Second, the lack of a set time limit for corneal topography detection may result in dynamic changes in the pupil diameter throughout the day. Third, the pupillary PSZ area may not accurately represent the summed corneal power shift. For example, individuals with a higher initial SE may achieve a greater summed corneal power shift than the individuals with a lower initial SE [15].

### Advantages and limitations of this study

This study presents several improvements over prior research. We employed a more realistic approach to describe and compute corneal reshaping parameters, enhancing the credibility of our conclusions. Additionally, we introduced novel measurements of pupillary PSZ area, TZ fractal dimension, and radius ratio, as well as evaluated their impact on ALG. Despite these strengths, it is essential to acknowledge the study’s limitations. First, the sample size was limited; a larger sample size would enhance the study’s robustness. Second, this study focused solely on corneal topography, neglecting potential impacts on visual quality such as ocular wavefront or contrast sensitivity function associated with larger eccentric distances and smaller TZ areas.

## Conclusion

In conclusion, IPP software enabled detailed delineation of corneal topography and extraction of comprehensive measurement parameters. Lens designs resulting in larger eccentric distances or smaller TZ areas may offer improved myopic control efficacy.

## Data Availability

The datasets used and/or analyzed during the current study are available from the corresponding author upon reasonable request.

## List of abbreviations

ALG: Axial length growth
TZ: Treatment zone
PSZ: Peripheral steepened zone
IPP: Image-Pro Plus
SE: Spherical equivalent

## References

[1] Jonas JB, Ang M, Cho P, Guggenheim JA, He MG, Jong M, Logan NS, Liu M, Morgan I, Ohno-Matsui K (2021). IMI Prevention of Myopia and its progression. Invest Ophthalmol Vis Sci.

[2] Morgan IG, French AN, Ashby RS, Guo X, Ding X, He M, Rose KA (2018). The epidemics of myopia: Aetiology and prevention. Prog Retin Eye Res.

[3] Sun J, Zhou J, Zhao P, Lian J, Zhu H, Zhou Y, Sun Y, Wang Y, Zhao L, Wei Y (2012). High prevalence of myopia and high myopia in 5060 Chinese university students in Shanghai. Investig Ophthalmol Vis Sci.

[4] Huang J, Wen D, Wang Q, McAlinden C, Flitcroft I, Chen H, Saw SM, Chen H, Bao F, Zhao Y (2016). Efficacy comparison of 16 interventions for Myopia Control in Children: A Network Meta-analysis. Ophthalmology.

[5] Cho P, Cheung SW, Edwards M (2005). The longitudinal orthokeratology research in children (LORIC) in Hong Kong: a pilot study on refractive changes and myopic control. Curr Eye Res.

[6] Hiraoka T, Kakita T, Okamoto F, Takahashi H, Oshika T (2012). Long-term effect of overnight orthokeratology on axial length elongation in childhood myopia: a 5-year follow-up study. Investig Ophthalmol Vis Sci.

[7] Lin W, Gu T, Bi H, Du B, Zhang B, Wei R (2022). The treatment zone decentration and corneal refractive profile changes in children undergoing orthokeratology treatment. BMC Ophthalmol.

[8] Chen MF, Liu XT, Zhang F, Wang YL, Mao XJ (2022). The influencing factors and the effect of myopia control in children treated with orthokeratology. Zhonghua Yan Ke Za Zhi.

[9] Vincent SJ, Cho P, Chan KY, Fadel D, Ghorbani-Mojarrad N, González-Méijome JM, Johnson L, Kang P, Michaud L, Simard P (2021). CLEAR - orthokeratology. Contact lens Anterior eye: J Br Contact Lens Association.

[10] Villa-Collar C, Carracedo G, Chen Z, Gonzalez-Méijome JM (2019). Overnight Orthokeratology: Technology, Efficiency, Safety, and Myopia Control. J Ophthalmol.

[11] Hiraoka T (2022). Myopia control with Orthokeratology: a review. Eye Contact Lens.

[12] Guo B, Wu H, Cheung SW, Cho P. Manual and software-based measurements of treatment zone parameters and characteristics in children with slow and fast axial elongation in orthokeratology. Ophthalmic Physiological Optics: J Br Coll Ophthalmic Opticians (Optometrists) 2022. 10.1111/opo.12981 35366332

[13] Yang X, Bi H, Li L, Li S, Chen S, Zhang B, Wang Y (2021). The Effect of relative corneal refractive power shift distribution on axial length growth in myopic children undergoing Orthokeratology Treatment. Curr Eye Res.

[14] Hu ZYCZDYYZYH (2020). Effect of eccentricity of overnight orthokeratology lenses on axial growth and visual quality. Int Eye Sci.

[15] Hu Y, Wen C, Li Z, Zhao W, Ding X, Yang X (2019). Areal summed corneal power shift is an important determinant for axial length elongation in myopic children treated with overnight orthokeratology. Br J Ophthalmol.

[16] Santodomingo-Rubido J, Villa-Collar C, Gilmartin B, Gutiérrez-Ortega R (2013). Factors preventing myopia progression with orthokeratology correction. 1538–9235.

[17] Mei Y, Tang Z, Li Z, Yang X. Repeatability and Reproducibility of Quantitative Corneal Shape Analysis after Orthokeratology Treatment Using Image-Pro Plus Software. *Journal of ophthalmology* 2016, 2016:1732476. 10.1155/2016/1732476 PMC5059590 27774312

[18] Lin W, Li N, Gu T, Tang C, Liu G, Du B, Wei R (2021). The treatment zone size and its decentration influence axial elongation in children with orthokeratology treatment. BMC Ophthalmol.

[19] Pauné J, Fonts S, Rodríguez L, Queirós A. The role of back Optic Zone Diameter in Myopia Control with Orthokeratology lenses. J Clin Med 2021, 10(2). 10.3390/jcm10020336 PMC7831104 33477514

[20] Hu Y, Yu J, Cui X, Zhang Z, Li Q, Guo W, Zhao C, Chen X, Meng M, Li Y (2021). Combination usage of AdipoCount and Image-Pro Plus/ImageJ Software for quantification of adipocyte sizes. Front Endocrinol (Lausanne).

[21] Lian KM, Lin T (2021). Value of image-pro plus for assisting virtual touch tissue imaging in the diagnosis of thyroid nodules. Clin Hemorheol Microcirc.

[22] Lemmens S, Devulder A, van Keer K, Bierkens J, Boever Pd, Stalmans I (2020). Systematic review on Fractal Dimension of the Retinal vasculature in Neurodegeneration and Stroke: Assessment of a potential biomarker. Front NeuroSci.

[23] Lennon FE, Cianci GC, Cipriani NA, Hensing TA, Zhang HJ, Chen CT, Murgu SD, Vokes EE, Vannier MW, Salgia R (2015). Lung cancer-a fractal viewpoint. Nat Reviews Clin Oncol.

[24] Kakita T, Hiraoka T, Oshika T (2011). Influence of overnight orthokeratology on axial elongation in childhood myopia. Investig Ophthalmol Vis Sci.

[25] Zhang Z, Chen Z, Chen Z, Zhou J, Zeng L, Xue F, Qu X, Zhou X (2022). Change in corneal power distribution in Orthokeratology: a predictor for the change in axial length. Transl Vis Sci Technol.

[26] Xu XL, Lin X, Zhao LH, Cai T, Du XL (2023). Long-term prevention and control effects of orthokeratology lenses designed for small treatment zones on children and adolescents with myopia. Zhonghua Yan Ke Za Zhi.

[27] Cho P, Cheung S-W (2012). Retardation of myopia in Orthokeratology (ROMIO) study: a 2-year randomized clinical trial. Investig Ophthalmol Vis Sci.

[28] Wang D, Wen D, Zhang B, Lin W, Liu G, Du B, Lin F, Li X, Wei R. The Association between Fourier Parameters and clinical parameters in myopic children undergoing Orthokeratology. Curr Eye Res 2021:1–9. 10.1080/02713683.2021.1917619 34096430

[29] Kang P, Swarbrick H (2011). Peripheral refraction in myopic children wearing orthokeratology and gas-permeable lenses. Optom Vis Sci.

[30] Guo B, Cheung SW, Kojima R, Cho P (2021). One-year results of the variation of Orthokeratology Lens Treatment Zone (VOLTZ) study: a prospective randomised clinical trial. Ophthalmic Physiological Optics: J Br Coll Ophthalmic Opticians (Optometrists).

[31] Xu Y, Deng J, Zhang B, Xu X, Cheng T, Wang J, Xiong S, Luan M, Zou H, He X (2023). Higher-order aberrations and their association with axial elongation in highly myopic children and adolescents. Br J Ophthalmol.

[32] Faria-Ribeiro M, Navarro R, González-Méijome JM (2016). Effect of pupil size on Wavefront Refraction during Orthokeratology. Optom Vis Sci.

[33] Hiraoka T, Kakita T, Okamoto F, Oshika T (2015). Influence of ocular wavefront aberrations on axial length elongation in myopic children treated with overnight Orthokeratology. Ophthalmology.

[34] Hiraoka T, Mihashi T, Okamoto C, Okamoto F, Hirohara Y, Oshika T (2009). Influence of induced decentered orthokeratology lens on ocular higher-order wavefront aberrations and contrast sensitivity function. J Cataract Refract Surg.

[35] Chen J, Huang W, Zhu R, Jiang J, Li Y (2018). Influence of overnight orthokeratology lens fitting decentration on corneal topography reshaping. Eye Vis (London England).

[36] Santodomingo-Rubido J, Villa-Collar C, Gilmartin B, Gutierrez-Ortega R, Suzaki A (2015). The effects of entrance pupil centration and coma aberrations on myopic progression following orthokeratology. Clin Experimental Optometry.

[37] Chen Z, Niu L, Xue F, Qu X, Zhou Z, Zhou X, Chu R (2012). Impact of pupil diameter on axial growth in orthokeratology. 1538–9235.

[38] Downie LE, Lowe R (2013). Corneal reshaping influences myopic prescription stability (CRIMPS): an analysis of the effect of orthokeratology on childhood myopic refractive stability. Eye Contact Lens.

